# Foreign Summary

**Published:** 1839-12-14

**Authors:** 


### FOREIGN SUMMARY.

*Removal of an Enormous Fibrous Tumour of the Uterus,* By M. Scoutetten.—Madame R., a lady of easy circumstances, at Metz, is forty years of age, strong, and of good constitution; she has had several children, and her labours have always been unattended by any bad effect. In 1834 her catamenia were disturbed; afterwards abundant but easily repressed hæmorrhages supervened, without any evident cause; and they were accompanied with sensibility in the epigastrium, and of derangement in the digestive functions.

Notwithstanding the evident progress of the disease, and the increasing swelling of the abdomen, the patient was but little distressed, when suddenly, in April 1837, she was attacked with a considerable hæmorrhage, which she attributed to fatigue. She went to bed, and a few instants after she felt a shock, followed by pains like those of labour, which increased and succeeded each other rapidly. An accoucheur was called, who discovered by the touch a very long tumour engaged in the neck of the uterus. The patient remained two days in a state of horrible suffering; it was hoped that nature might be able alone to relieve her. The seriousness of the disease and the imminence of the danger suggested a consultation, and I was called in. I found, by an attentive examination, that the tumour was hard, resisting, and smooth on its surface, and had the principal external characters of a fibrous tumour.

The patient was placed on the edge of a bed, and her legs and thighs being separated and fixed, I introduced my fingers in the vagina in the hope that they might be sufficient to draw the tumour out, but I soon found that this was impossible. I then took strong toothed forceps; they tore the surface of the tumour, but could not move it. I tried to pass a thread round the tumour with a *serre-nœud*, but the ligature slipped, and fell off directly. I then introduced a long and narrow forceps cautiously into the cavity of the uterus, and with them the tumour was seized and slowly drawn out; I found that it was fixed by a large surface to the interior of the uterus, and that that organ was inverted. Was I now to divide the attachment with the bistoury? I should so expose myself to a hæmorrhage that might be fatal. Was it not more prudent to confine myself to the application of a ligature without endeavouring for the present to return the uterus? I knew that I might thus excite inflammation of that organ and peritonitis; but I preferred to expose myself to combat an inflammation rather than a hæmorrhage that might be at once destructive.

I therefore put a strong but fine ligature on the tumour at its attachment; it was drawn tight and the tumour almost immediately became brown, which I attributed to the arrest of the blood in the numerous vessels that traversed it. I surrounded it with a compress covered with cerate, and placed the whole between the thighs of the patient.

During the first day the abdomen was tense; but only slightly painful, the pulse scarcely febrile. I ordered only a friction of oil on the abdomen and the application of a cataplasm. On the next day, the patient suffered a little more, but there was no serious symptom. On the third the weight of the tumour had lengthened the cellular bands which united it to the uterus nearly half an inch, and it was now easy to see that they might without inconvenience be divided with the knife; I did so at once: the tumour was removed, the uterus gently pushed back, and all the bad symptoms disappeared.

Twelve days after, the patient was so far recovered that she could go out and walk; and since that time her health has never been a moment deranged. The tumour weighed 35 ounces; it was ovoid; its great circumference measured 16½ inches, its middle circumference 12 inches and five lines; its tissue was composed of concentric fibres.—*Lond. from Paris Med. Gaz.*

---

*Gangrene resulting from the employment of a Starched Bandage in a case of Fracture of the Patella.* By Dr. Defer, of Metz.—A man, æt. 40, received a transverse fracture of the patella, from a fall. The medical officer who was called in, applied first a bandage, to bring the portions of bone together, and then a starched roller, which extended from the toes to the upper third of the thigh; the limb was then placed on an inclined plane. The patient was occasionally visited; but, as he suffered scarcely any pain, the apparatus was not altered. About six weeks after the accident his attendant, on proceeding to remove the apparatus, found the smell such as to induce the presumption that gangrene had supervened; and Dr. Defer was called in. He found the odour that exhaled from the limb such as could not for an instant permit a doubt of the existence of gangrene. The toes which the roller had not covered were mummified and completely insensible. On removing the bandage, the gangrene was seen to extend to within seven inches of the knee. The foot was cold and insensible, and the epidermis had begun to separate. The ankle-joint was exposed; the ligaments were destroyed, and the tendons, being no longer restrained, were extended like cords. The bones of the leg were also exposed in their lower third, and the tendons had begun to slough. Amputation was performed, and the patient recovered.

[The practice in this case was shameful; but the error was less in the employment of a particular bandage than in the subsequent inattention to the progress of the injured part. Any other bandage would probably have produced the same effects, when applied immediately after the accident, and suffered to remain for six weeks.]—*Gaz. Med. from Brit. and For. Med. Rev.*

---

*On Congenital Dislocations of the Femur.* By M. Bouvier.—At the meeting of the Academy of Medicine, on the 16th of April, M. Bouvier presented three specimens of this rare malformation. One of them was from a woman, æt. 26, who had died of phthisis. Both femurs were dislocated. On the right side the head of the femur was still attached by a portion of the capsular ligament, which lay between the head and the outer surface of the ilium. The cotyloid cavities were reduced in size, and triangular; and, notwithstanding the wasting of the heads of the femur, the cavities were both too small to admit them.

The second case was one of double dislocation, in a woman, aged 29; but M. B. had received only the left hip-joint, and that, like those of the preceding subject, in an imperfect state. In the place of the cotyloid cavity there was only a slight superficial triangular depression, quite incapable of receiving the head of the femur, though it was much atrophied. The capsule was lengthened, and, as in the preceding case, interposed between the femur and the dorsum of the ilium. It was dilated at its extremities, but contracted, like an isthmus, at the middle, so that the head of the femur could not, during life, have been made to pass through it.

In the third case the left femur only was dislocated. The patient had been a porter, lame from birth, but robust. The head of the femur was higher and more forward than in the other cases. Enveloped above by its capsular ligament, it was placed on a small false articulating surface, on the ilium, to the edge of which the ligament was attached. The muscles attached to the upper part of the femur were all healthy, except the quadratus, which was atrophied, and in part fatty. On trying to draw down the head of the femur, by pulling it parallel to the axis of the body, and at the same time pushing up the ilium in the opposite direction, an insurmountable resistance was found to arise, from the tightness of the anterior and inner part of the capsule. But when the femur was strongly flexed and at the same time rotated very much outwards, and in this position (as in M. Desprez's mode of reduction) drawn parallel to the axis of its body, the head could easily be placed opposite to the acetabulum, though, from the contracted size and form of that cavity, it could not be placed in it.

[In reference to the important but still problematical question of the reducibility of congenital dislocations of the femur, these cases would show that the chief points to be considered are, the more or less complete obliteration of the cotyloid cavity, the contraction of the capsular ligament between the acetabulum and the part which surrounds the neck of the femur, the extreme resistance which the anterior and internal part of the capsule presents to any efforts at elongation of the limb, the difficulty of moving the thigh during life into the position in which, after death, a kind of reduction may be effected; and, lastly, the difficulty of retaining the limb during extension in the reduction which has been effected by its flexion.]--*Brit. and For. Med. Rev.*, *from Bulletin de l'Acad. Roy. de Méd. Juin* 30, 1839.

---

*Singular Case of a Woman delivered of Five Children.*—Giuseppa Califani, of Naples, at the age of fourteen years and three months, was married to a man aged twenty-seven, by whom she had ten children at eight accouchements; at the fifth and sixth producing twins. She lived with her husband ten years, and remained a widow three years after his death; she then took a second husband, whose age was about twenty-nine. After two regular accouchements, upon her third pregnancy she became enormously large; so that, at seven months, she appeared to be at the termination of her natural period. She was taken, however, at seven months, with labour-pains, and brought forth successively, and by natural presentations, five living children, all of whom were baptized. The mother did not suffer any thing extraordinary. Four of these children were females, and one male. The male infant was delivered first, and, after a few minutes, one female; then, after a cessation of fifteen minutes' interval between each, the other three followed. The infants much resembled each other, and were of a regular form, and well grown, and very nearly of the ordinary size of a seven months' fœtus; each weighed about three and a half pounds, and measured in length a French foot. The insertion of the umbilical cord was about four lines lower down than ordinarily. The placentas, with their membranes, were four instead of five; and each had its proper umbilical cord, except the fourth, which contained two in one large sac. The fœtus, with their membranes, placenta, and umbilical cords, are preserved in the Royal Anatomical Museum of the University of Naples. Vincenzo Licci, of Calimera, in Otranto; Vincenzo Massari, of Molfetta, Brian ; and Dr. Antonio Scacani of Naples, conducted the examination.—*Ib., from Bulletino delle Scienze Mediche. Agosto e Settembre*, 1838.

---

*New Test for the Detection of Pregnancy.*—(L'Experience, July 25, 1839.)—M. Nauche found that the urine of pregnant women contains a particular substance, which, when the urine is allowed to stand, separates and forms a pellicle on the surface. M. Eguiser, from an extensive series of observations, has confirmed this fact, and found that the *kisteine*, as this particular substance has been called, is constantly formed on the surface of the urine of women in a state of pregnancy.

The urine must be allowed to stand for from two to six days, when minute opaque bodies are observed to rise from the bottom to the surface of the fluid, where they gradually agglomerate and form a continuous layer over the surface. This layer is so consistent that it may be almost lifted off by raising it by one of its edges. This is the kisteine. It is whitish, opalescent, slightly granular, and can be compared to nothing better than to the fatty substance which swims on the surface of soups, after they have been allowed to cool. When examined by the microscope it has the aspect of a gelatinous mass, without determinate form ; sometimes cubical shaped crystals are discovered on it, but this appearance is only observed when it has stood for a long time, and are to be regarded as foreign to it. The kisteine remains on the surface for several days ; the urine then becomes turbid, and small opaque masses become detached from the kisteine, and fall to the bottom of the fluid ; and the pellicle soon becomes destroyed.

The essential character of the urine of pregnancy, then, is the presence of kisteine; and the characters of the pellicle are so peculiar that it is impossible to mistake it for any thing else. A pellicle sometimes forms on the surface of the urine of patients labouring under phthisis, abscess, or catarrh of the bladder, but may be easily distinguished by this circumstance, that it does not form in such a short time as the kisteine, and that, in place of disappearing, as this last, in a few days, it increases in thickness, and at last is converted into a mass of mouldiness. There exists, likewise, a very marked difference between its mucous aspect and that of kisteine—a difference which it is difficult to describe, but which is easily recognized.

*Kisteine* appears to exist in the urine from the first month of pregnancy till delivery. M. Rousseau has even recognized it in the urine of a few gravid animals.—*Edinburgh Medical and Surgical Review.*

---

*Clot Bey.*—Clot Bey, first physician to Mehemet Ali, has lately been created, by the Pope, Commander of the Order of Gregory the Great.--*Med. Gaz.*

